# A numerical study of the relevance of the electrode-tissue contact area in the application of soft coagulation

**DOI:** 10.1038/s41598-025-13836-0

**Published:** 2025-07-31

**Authors:** Christoph Busch, Stefan J. Rupitsch, Knut Moeller

**Affiliations:** 1 Institute of Technical Medicine (ITeM), Furtwangen University, Villingen-Schwenningen, 78054 Germany; 2 Department of Microsystems Engineering, Faculty of Engineering, University of Freiburg, Freiburg, 79110 Germany

**Keywords:** HF soft coagulation, Finite element modeling, Electrode-tissue contact surface, Biomedical engineering, Computational science

## Abstract

Monopolar electrocoagulation is a well-established surgical technique to achieve hemostasis by selectively destroying biological tissue through the application of high-frequency alternating current. However, this technique is associated with unwanted tissue damage. In this context, computational simulation is a valuable tool that can improve our understanding of such complex processes and highlight important application parameters in the direction of an improved control function to achieve safer and more reliable results. Despite its critical role in surgical applications, the influence of the electrode-tissue contact area has received little to no attention in previous simulation studies. To address this gap, the present study investigates the sensitivity of temperature distribution and necrotic volume formation to variations in electrode-tissue contact area. For this purpose, a multiphysics finite element model was developed to simulate HF current induced soft coagulation using a ball electrode under varying contact areas. Our findings demonstrate that variations in the contact area significantly impact temperature development and, consequently, necrosis formation. These results highlight the crucial role of the contact area in the electrocoagulation process and its associated necrosis formation. Furthermore, it was observed that when the boiling point of water is reached inside the tissue, complete necrosis has not yet formed at the contact site, which could lead to further undesired effects. Consequently, it is essential to consider the contact area in computational simulations and the development of novel control features for safer and more reliable electrocoagulation.

## Introduction

There is no longer a necessity to speculate about the contemporary relevance of high-frequency (HF) surgery across a diverse range of surgical applications, as its advantages have been well-established and widely recognized for many decades^1^. As with any surgical intervention, the procedure must be both effective and safe despite human factors and patient variability. Nevertheless, unwanted tissue damage like nerve irritation or axonal damage is more likely for monopolar than for bipolar applications^2–4^, since the current flows through a larger part of the patient’s body. Additionally, the energy deposition into the tissue could vary through diverse factors like an unfavorable power setting or control algorithm at the HF generator, changing tissue properties, or simply changes through user instrument handling. For this reason, the development of medical devices featuring control applications is of great interest in order to control as many influencing parameters as possible and thus ensure more robust and reliable surgical outcomes.

A control application that precisely defines the volume and shape of tissue necrosis could play a crucial role in mitigating unwanted and unexpected complications. By minimizing the risk of unintended tissue damage, such an application would significantly enhance both the safety and efficacy of the procedure. The development of such control features requires a comprehensive understanding of the application, as well as the identification of the most critical and influential parameters necessary to achieve the desired surgical outcome. In this regard, computational modeling and simulation serve as a powerful tool, not only for evaluating the performance of novel medical devices in a virtual environment but also for elucidating complex biophysical phenomena, thereby deepening the understanding of the underlying mechanisms of the application. This highlights the pressing need for further investigation into these factors and their potential impact on procedural outcomes. A more profound understanding of these influences could contribute to the refinement of existing technologies, the optimization of control strategies, and the overall advancement of electrosurgical applications.

Nevertheless, it is crucial to emphasize that an appropriate modeling approach must accurately and reliably represent real-world physical processes with sufficient precision. For this reason, a thorough understanding of the fundamental mechanisms underlying HF-induced tissue heating and necrosis prediction is essential. HF alternating currents, typically ranging between 200 kHz and 5 MHz, are utilized to achieve a variety of effects, including tissue cutting, coagulation, or a combination of both^5^. During this process, the application of HF electrical current results in heating at the cellular level. This thermal phenomenon can primarily be attributed to Joule heating, which occurs due to the conversion of electrical energy into heat within the tissue. Joule heating is a fundamental process wherein the flow of electrical current through a conductor – in this case, biological tissue – generates heat as a result of the resistance encountered by the current flow. This effect is a key aspect of HF modeling and plays a critical role in predicting thermal cell death.

In our study, we focus on the conventional HF application monopolar soft coagulation using a ball electrode, wherein a pure sinusoidal HF current is employed to achieve precise tissue hemostasis, which refers to the controlled cessation of bleeding during surgical interventions. The deliberate application of thermal energy to the targeted tissue induces denaturation of structural proteins and desiccation of both intra- and extracellular water. These effects collectively contribute to necrosis formation in the treated tissue and to achieve hemostasis. The importance of this method is particularly evident in surgical interventions where the primary objective is to manage hemorrhage effectively.

Modeling approaches are frequently employed in specialized HF applications to accomplish various objectives. Generally, these approaches aim to optimize surgical procedures^6–10^, enhance safety for both users and patients^11–13^, and facilitate a deeper understanding of the complex processes and interactions that occur when HF electrical currents interact with biological tissue^14–16^. However, the modeling of HF applications is basically dependent on the specific application being studied. This is because differences exist depending on the application type and its methodology, which must always be carefully taken into account^17^. Numerous modeling approaches are specifically tailored to address the requirements of radiofrequency (RF) ablation^8,14–16,18^ or vessel sealing^6,7,11–13^. While these applications differ fundamentally in their methodologies, all of the corresponding models share a common feature, the utilization of the finite element method (FEM) to simulate temperature distribution by applying an electric field to the tissue under investigation.

To the best of our knowledge, the influence of the electrode-tissue contact area has not yet been adequately addressed in the existing literature, despite its major importance in this application. This is likely because in other applications, such as tumor ablation, this factor is often considered to have minimal impact due to the well-defined and consistent contact area determined by the electrode’s geometry. For applications such as bipolar coagulation, the research group of Dodde et al. (2008)^12^ showed that variations in forceps jaw geometry can significantly affect temperature distribution. However, even in this application, the contact area can be assumed to remain constant during the application and is primarily defined by the instrument’s design. Nevertheless, this assumption is invalid for standard monopolar HF applications, such as soft coagulation through a ball electrode. The contact area of the electrode with the tissue varies as a result of the user’s handling. Therefore, it directly affects the deposition energy and the subsequent development of tissue temperature.

While Karaki et al. (2017)^19^ demonstrated in their study for a monopolar coagulation process that varying contact areas, achieved through different electrode diameters, can influence temperature development, the implications for temperature dynamics and resulting necrosis formation have not been analyzed and discussed. Therefore, the effect of varying contact areas between the active electrode and biological tissue in monopolar soft coagulation remains insufficiently investigated for usage in developing a control application. Therefore, the research question towards knowledge for developing a future control application in an HF generator can be phrased to: How does the contact area between active electrode and tissue in a monopolar soft coagulation influence the temperature dynamics with subsequent necrosis formation?

In order to formulate a response to the research question, it is necessary to investigate the sensitivity of the temperature dynamics and the development of the necrotic tissue volume to variations in the electrode-tissue contact areas. To this end, well-established physical equations are used in a multiphysics modeling approach to investigate the effects of the contact area on the thermal behavior during monopolar soft tissue coagulation. The objective of this research is to gain further important insights into the electrosurgical monopolar soft coagulation process. The findings can be regarded as a modest yet pivotal aspect that contributes to a more profound comprehension of the application and has the potential to enhance the safety and efficacy of future electrosurgical systems.

## Methods

To investigate the research question, a multiphysical finite element (FE) simulation model was developed in COMSOL Multiphysics®(2023)^20^, based on fundamental physical equations. This model simulates the application of monopolar ex vivo tissue soft-coagulation using a ball electrode with a diameter of 4 mm. Due to the specific nature of the application, symmetry considerations were utilized from the outset to effectively reduce the complexity of the model. Consequently, the originally three-dimensional (3D) application was simplified into a two-dimensional (2D) axisymmetric FE model. This dimensional reduction resulted in a significant decrease in model size, thereby substantially reducing the degrees of freedom in the computational problem. The corresponding assumptions for the physical representation of the 3D application in a 2D axisymmetric FE model are outlined in detail in the subsequent subsections.

The developed model incorporates essential mechanical, thermal, and electrical phenomena by accounting for thermo-mechanical and thermo-electrical coupled interactions. The simulation represents the application process dynamically over a three-second period. During the first second, the active electrode is displaced, leading to both physical contact and thermal exchange between the electrode and the biological tissue, which depends on the contact area. After the electrode displacement, a voltage is applied to the active electrode for two seconds (from the first to the third second). This induces Joule heating in the tissue, which subsequently leads to thermal tissue damage. The simulations were performed on a workstation equipped with an AMD Ryzen Threadripper PRO 5995WX 64-core processor and 512 GB of RAM. Following the simulations, the relevant simulation results were exported, imported into MATLAB^21^, analyzed, and visualized graphically.

The following sections elaborate on the underlying physical phenomena and the associated system of equations that form the foundation of the simulation model. Furthermore, all physical assumptions regarding materials, boundary conditions, meshing strategies, and computational settings within the FE model are described in detail.

### General description of the physical problem

When modeling a mechanically, thermally, and electrically coupled problem, as encountered in the present study, it is necessary to consider both the equation of motion, which is derived from the conservation of momentum, and the heat conduction equation. From the perspective of continuum mechanics, the kinematic description of a material point transitioning from its reference position to its deformed position can be expressed using the deformation gradient $\textbf{F}$, given by $\textbf{F}=\partial \textbf{x}/\partial \textbf{X}=\textbf{I}+\nabla \textbf{u}$. Here, $\textbf{x}$ represents the current deformed position of the material point, $\textbf{X}$ denotes the reference position, $\textbf{I}$ is the identity tensor, and $\nabla \textbf{u}$ is the displacement gradient vector.

The ball electrode was modeled as a linear elastic material, while the biological soft tissue was assumed to be hyperelastic, homogeneous, isotropic, and incompressible liver tissue^22–25^. Since the objective of this analysis is to provide a qualitative representation of the contact area of different sizes between the electrode and the biological tissue for Joule heating, the tissue’s mechanical behavior was approximated using the Neo-Hookean material model. This model is widely used in biomechanical simulations due to its low computational complexity and the limited number of required material parameters^22,24^. In this context, it was observed to provide stable and convergent numerical solutions within the applied simulation framework.

The assumption of tissue incompressibility implies that the tissue volume remains constant during deformation, which corresponds to an isochoric process. This condition is enforced by the volume ratio $J=\det (\textbf{F}) = 1$. To account only for the isochoric part of the deformation, the strain energy density function $W_{\textrm{s}}$ is expressed in terms of the modified first invariant $\bar{I}_1$, defined as $\bar{I}_1=J^{-2/3}\,\textrm{tr}(\textbf{C})=J^{-2/3}\,\textrm{tr}(\textbf{F}^\intercal \textbf{F})$. Thus, $W_{\textrm{s}}$ for the incompressible Neo-Hookean material reads

1 $$\begin{aligned} W_{\textrm{s}} = \frac{\mu }{2}(\bar{I}_{1} - 3), \end{aligned}$$

where $\mu$ denotes the second Lamé parameter, also called shear modulus of the material, which is defined by $\mu =E/(2(\nu +1))$, with *E* and $\nu$ being Young’s modulus and Poisson’s ratio, respectively. In the strictly incompressible case $J=1$, we have $\bar{I}_{1}=I_{1}$, but the isochoric formulation ensures consistent modeling also in numerical implementations that approximate incompressibility.

The equation of motion describes the acceleration of a small volume of material resulting from the divergence of internal stresses and the influence of external body forces. In the reference configuration, the equation is expressed in Lagrangian form as

2 $$\begin{aligned} \rho _0 \frac{\partial ^2 \textbf{u}}{\partial t^2}= \nabla \cdot (\textbf{F}\textbf{S})^\intercal + \textbf{F}_{\textrm{ext}}, \end{aligned}$$

where $\rho _0$ is the material density in the reference configuration, *t* denotes the time, $\textbf{S}$ is the second Piola-Kirchhoff stress tensor, and $\textbf{F}_{\textrm{ext}}$ represents the external body forces. Since we assume idealized conditions in which the displacement of the electrode and its resulting contact with the biological tissue occurs slowly in small incremental steps, it can be assumed that a quasi-static equilibrium state exists at all times under the investigated conditions. Consequently, the inertial term on the left-hand side of the equation of motion can be neglected by setting

3 $$\begin{aligned} \rho _0\frac{\partial ^2 \textbf{u}}{\partial t^2}=0. \end{aligned}$$

This assumption implies that inertial forces are insignificant in this context. External body forces, such as gravity, are likewise not considered, as the simulation is conducted under idealized and static conditions. Furthermore, the electrosurgical soft coagulation process typically occurs within a few seconds. As a result, gravitational effects, such as fluid displacements or thermally induced convection, are expected to be minimal, as they develop too slowly to exert a substantial influence during the short application time. However, it should be noted that these assumptions may not be valid in dynamic scenarios or models where gravity acts at oblique angles relative to the tissue surface. Under these simplifying assumptions, Equation (2) reduces to the static form

4 $$\begin{aligned} 0= \nabla \cdot (\textbf{F}\textbf{S})^{\intercal }. \end{aligned}$$

The relationship between the equation of motion and the hyperelastic material is established via the second Piola-Kirchhoff stress tensor

5 $$\begin{aligned} \textbf{S}= 2\frac{\partial W_{\textrm{s}}}{\partial \textbf{C}}. \end{aligned}$$

For the selected hyperelastic material model, the stress tensor is derived from the strain energy density function. However, the incompressibility constraint of the tissue ($J=1$) necessitates the introduction of the pressure *p* as a Lagrange multiplier in the numerical implementation^26^. Incorporating the Neo-Hookean material model from Equation (1), the second Piola-Kirchhoff stress tensor is expressed as

6 $$\begin{aligned} \textbf{S}= \mu \textbf{I}-p\textbf{C}^{-1}. \end{aligned}$$

This formulation ensures the enforcement of the incompressibility condition^26^.

At the interface between the electrode and the tissue, a frictionless contact is assumed. This assumption is justified by the controlled and quasi-static nature of a generalized electrode placement during the electrosurgical monopolar soft coagulation procedure, where the electrode is pressed perpendicularly into the tissue without lateral motion. As a result, no tangential displacement occurs at the contact interface, and thus no shear stresses or frictional forces are generated. Furthermore, adhesive effects between the electrode and the liver tissue are considered negligible as a result of an idealized smooth electrode surface. Under these conditions, the contact forces are entirely conservative, meaning that they do not contribute to mechanical energy dissipation. Consequently, no non-conservative contact terms arise in the mechanical or thermal energy balance, and the internal energy changes are solely attributed to elastic deformation and resistive Joule heating.

The thermo-mechanical and thermo-electrical coupled problem can be described based on the principle of energy conservation. Using the heat conduction equation, the energy conservation equation in its general form is expressed as

7 $$\begin{aligned} \rho C_{\textrm{p}} \frac{\partial T}{\partial t} =\nabla \cdot (k \nabla T) + Q_{\textrm{el}} + Q_{\textrm{mech}}, \end{aligned}$$

where $\rho$ denotes the material density, $C_{\textrm{p}}$ the specific heat capacity, *T* the temperature, *t* the time, *k* the thermal conductivity of the medium, $Q_{\textrm{el}}$ the externally supplied electrical energy, and $Q_{\textrm{mech}}$ the energy contribution from mechanical deformation.

Since the model considers ex-vivo tissue, typical terms from^27^ proposed bio-heat equation, such as the metabolic heat generation or cooling at the application site due to capillary blood flow, are not included. To further simplify the problem, the thermal expansion of the materials is neglected, and the coefficient of thermal expansion $\alpha$ is assumed to be zero. Additionally, as the deformation of the tissue is assumed to be purely elastic and incompressible, no irreversible dissipation occurs, and therefore no heat is generated by mechanical deformation. Consequently, the term $Q_{\textrm{mech}}$ is omitted from Equation (7).

The energy input $Q_{\textrm{el}}$ into the biological tissue, resulting from the applied voltage $V_{\textrm{sc}}$, is attributed to Joul heating and is defined by the scalar product of the current density $\textbf{J}$ and the electric field $\textbf{E}$. Considering the frequencies of the applied alternating current, which is approximately 350 kHz in monopolar soft-coagulation, it is common to simplify the electrical problem by treating it as quasi-electrostatic^28^. In this context, the applied voltage is represented by the root-mean-square (RMS) value, and displacement currents are neglected, justifying a quasi-static approach. The electric potential can then be determined using the Laplace equation $\nabla \cdot \sigma \nabla V_\textrm{sc} = 0$, where $\sigma$ is the electrical conductivity of the material. Using Ohm’s law, the current density is given by $\textbf{J}=\sigma \textbf{E}$, where the electric field is defined as $\textbf{E}=-\nabla V_{\textrm{sc}}$. Electromagnetic losses are thus accounted for in Equation (7) through the heat source term $Q_{\textrm{el}}=\sigma |\nabla V_{\textrm{sc}}|^2$.

Heat transfer between the materials, particularly at the contact surface between the electrode and the tissue, is ensured using a Neumann boundary condition governed by Newton’s law of cooling. Given the relatively short application duration and the assumption that the temperature does not exceed 150 $^{\circ }\textrm{C}$, radiative heat transfer is neglected, setting the radiative heat transfer coefficient to zero^29^. As a result, the effective heat transfer coefficient *h* is determined solely by the thermal contact resistance $R_{\textrm{eq}}$, such that $h=R_{\textrm{eq}}^{-1}$. The thermal contact resistance $R_{\textrm{eq}}$ between the electrode and the tissue is assumed to be 100 $\mu \textrm{K}\textrm{m}^2/\textrm{W}$. Additionally, the Charron relationship is employed to account for the distribution of heat flux between the electrode and the tissue based on their material properties^29^. Consequently, the boundary condition for heat flux at the two interfaces electrode and tissue $\textbf{q}_{\textrm{et}}$, can be expressed as

8 $$\begin{aligned} \textbf{n}\cdot \textbf{q}_{\textrm{et}}=-k\frac{\partial T}{\partial \textbf{n}} =\frac{T_{\textrm{elec}}-T_{\textrm{tissue}}}{R_{\textrm{eq}}}, \end{aligned}$$

where $\textbf{n}$ is the normal vector to the respective interface, *k* represents the thermal conductivity of the respective material, $T_{\textrm{elec}}$ denotes the temperature at the electrode surface, and $T_{\textrm{tissue}}$ represents the temperature at the tissue surface.

To ensure full multiphysical coupling between the mechanical, thermal, and electrical effects, the electrical contact interaction between the electrode and the biological soft tissue was modeled using the Mikic elastic correlation^30^. This model introduces a pressure- and material-dependent contact conductance $h_{\textrm{c}}$, which was implemented via a Neumann boundary condition at the interface. The resulting current density at the electrode interface is defined as

9 $$\begin{aligned} \textbf{n}\cdot \textbf{J}_{\textrm{elec}}=h_{\textrm{c}}(V_{\textrm{elec}}-V_{\textrm{tissue}}), \end{aligned}$$

while, by current conservation and symmetry, the condition at the tissue interface is given by

10 $$\begin{aligned} \textbf{n}\cdot \textbf{J}_{\textrm{tissue}}=h_{\textrm{c}}(V_{\textrm{tissue}}-V_{\textrm{elec}}), \end{aligned}$$

where $\textbf{J}_{\textrm{elec}}$ and $\textbf{J}_{\textrm{tissue}}$ denote the electric current densities across the interface, and $V_{\textrm{elec}}$ and $V_{\textrm{tissue}}$ are the electric potentials in the respective domains.

The contact conductance $h_{\textrm{c}}$ was calculated using the Mikic correlation, as implemented in COMSOL Multiphysics^30–32^. This formulation relates contact conductance to the local contact pressure and the effective elastic properties of the contacting materials at the contact interface. Specifically, the correlation is given by

11 $$\begin{aligned} h_{\textrm{c}}=1.54\sigma _{\textrm{c}}\frac{m_{\textrm{asp}}}{\sigma _{\textrm{asp}}}\left( \frac{\sqrt{2}p_{\textrm{c}}}{m_{\textrm{asp}}E_{\textrm{c}}}\right) ^{0.94}, \end{aligned}$$

where $\sigma _{\textrm{c}}$ denotes the effective electrical conductivity at the interface, $m_{\textrm{asp}}$ is the average asperity slope, $\sigma _{\textrm{asp}}$ the average asperity height, $p_{\textrm{c}}$ the local contact pressure, and $E_{\textrm{c}}$ the effective elastic modulus of the contacting pair. Further theoretical background on the Mikic correlation and its application to contact conductance problems is available in the work of Muzychka and Yovanovich (2023)^32^. The surface roughness was parameterized using an average asperity slope of $m_{\textrm{asp}}=0.4$ and an average asperity height of $\sigma _{\textrm{asp}}=1~\mu \textrm{m}$. The interfacial electrical conductivity $\sigma _{\textrm{c}}$ was calculated as the harmonic mean of the electrical conductivities of the electrode and tissue, and is given by

12 $$\begin{aligned} \sigma _{\textrm{c}}=\frac{2\sigma _{\textrm{el}}\sigma (T)}{\sigma _{\textrm{el}}+\sigma (T)}, \end{aligned}$$

where $\sigma _{\textrm{el}}$ denotes the electrical conductivity of the electrode, and $\sigma (T)$ is the temperature-dependent electrical conductivity of liver tissue. The effective elastic modulus $E_{\textrm{c}}$, required in Equation (11), was calculated internally by COMSOL from the mechanical properties of the contacting materials as

13 $$\begin{aligned} \frac{1}{E_{\textrm{c}}}=\frac{1-\nu _{\textrm{el}}^{2}}{E_{\textrm{el}}}+\frac{1-\nu _{\textrm{t}}^{2}}{E_{\textrm{t}}}, \end{aligned}$$

where $\nu _{\textrm{el}}$, $\nu _{\textrm{t}}$ are the Poisson’s ratios, and $E_{\textrm{el}}$, $E_{\textrm{t}}$ are Young’s moduli of the electrode and tissue, respectively. The local contact pressure $p_{\textrm{c}}$ used in the Mikic correlation is computed by COMSOL during the nonlinear hyperelastic contact simulation. This contact problem was solved using the Augmented Lagrangian method with the fully coupled solution method^26^. All other boundary conditions not explicitly associated with the multiphysical interactions are described in a subsequent section.

### Material parameter

In addition to the previously described physical interactions, it is essential to represent the material parameters in the model accurately. This is particularly crucial when these parameters are dependent on the primary variable to be solved. Several research groups have emphasized the importance of temperature-dependent tissue parameters in computational simulations of current-induced heat generation in biological tissues^14,17,33^. Therefore, different modeling approaches were employed for various parameters in this study in order to represent them accurately.

In our investigation, as in many other simulation studies^28^, we modeled the thermal conductivity using a simple linear function defined as

14 $$\begin{aligned} k(T) = k_{\textrm{ref}} + k_1 (T - T_{\textrm{ref}}), \end{aligned}$$

where $k_{\textrm{ref}}$ represents the thermal conductivity of the tissue at the reference temperature ($T_{\textrm{ref}}=25~^{\circ }\textrm{C}$), and $k_1$ is the control coefficient regulating the increase or decrease in thermal conductivity^34^. Outside the defined temperature range ($T<20~^{\circ }\textrm{C}$ and $T>100~^{\circ }\textrm{C}$), a constant value of *k*(*T*) was assumed.

The electrical conductivity was modeled using a piecewise continuous function, adapted from the approach used to simulate electrical conductivity in vessel sealing processes published by Chen et al. (2013)^17^. Changes in $\sigma (T)$ were divided into two temperature ranges and defined as

15 $$\begin{aligned} \sigma (T)=\left\{ \begin{array}{ll} \sigma _{\textrm{ref}}[1+0.02(T-T_{\textrm{ref}})] \cdot W(T) & T<100~^{\circ }\textrm{C}\\ 0.01~\mathrm {S/m} & T\ge 100~^{\circ }\textrm{C}\\ \end{array} \right. , \end{aligned}$$

where $\sigma _{\textrm{ref}}$ is the electrical conductivity of the tissue at $T_{\textrm{ref}}=25~^{\circ }\textrm{C}$, and *W*(*T*) is the temperature-dependent relative water content of the tissue. At the boiling point of water, it was assumed that the electrical conductivity rapidly decreases due to the evaporation of tissue water, resulting in a high-resistance state characterized by an electrical conductivity of 0.01 S/m above $100~^{\circ }\textrm{C}$.

To ensure a smooth transition around the temperature threshold from $99~^{\circ }\textrm{C}$ to $100~^{\circ }\textrm{C}$, a continuous first derivative was applied to $\sigma (T)$. Additionally, to account for the gradual evaporation of tissue water beginning at approximately $80~^{\circ }\textrm{C}$, below $100~^{\circ }\textrm{C}$ $\sigma (T)$ was multiplied by a logistic function describing the relative tissue water content. This relative water content is temperature-dependent and expressed as

16 $$\begin{aligned} W(T) = \frac{W_{\textrm{ref}}}{1+\textrm{exp}(0.35(T-T_{\textrm{s}}))}+W_{\textrm{end}}, \end{aligned}$$

where $W_{\textrm{ref}}$ is the initial water content of the tissue, $W_{\textrm{end}}$ is the water content at the end of coagulation, and $T_{\textrm{s}}$ represents the boiling point of water ($100~^{\circ }\textrm{C}$). Based on Busch et al. (2023)^35^, an initial water content of 70% was assumed, with a final content of 0.1% estimated at the end of the coagulation process.

**Table 1:** Model material parameters.

Material parameter		Value	Unit	Source
Ball electrode (stainless steel)				
Poisson’s ratio	$\nu _{\textrm{el}}$	0.29		^36^
Young’s modulus	$E_{\textrm{el}}$	200	$\textrm{GPa}$	^36^
Density	$\rho _{\textrm{el}}$	7900	$\mathrm {kg/m^{3}}$	
Thermal conductivity	$k_{\textrm{el}}$	15	$\textrm{W}/(\textrm{m}\cdot \textrm{K})$	
Electrical conductivity	$\sigma _{\textrm{el}}$	1.37e6	$\textrm{S}/\textrm{m}$	
Biological soft-tissue (liver tissue)				
Poisson’s ratio	$\nu _{\textrm{t}}$	0.5		
Young’s modulus	$E_{\textrm{t}}$	1.98	$\textrm{kPa}$	^37^
Density	$\rho _{\textrm{t}}$	1079	$\mathrm {kg/m^{3}}$	^38^
Control coefficient	$k_1$	0.001161	$\textrm{W}/(\textrm{m} \cdot \textrm{K} \cdot \mathrm {^{\circ }C})$	^34^
Thermal conductivity	$k_{\textrm{ref}}$	0.52	$\textrm{W}/(\textrm{m}\cdot \textrm{K})$	^38^
Electrical conductivity	$\sigma _{\textrm{ref}}$	0.13	$\textrm{S}/\textrm{m}$	^38^
Effective heat capacity	$C_{\textrm{eff}}$	3540	$\textrm{J}/(\textrm{kg}\cdot \textrm{K})$	^38^
Frequency factor	$A_{\textrm{0}}$	$7.39\cdot 10^{39}$	$\textrm{s}^{-1}$	^39^
Activation energy	$E_{\textrm{a}}$	$2.577\cdot 10^{5}$	$\textrm{J}/\textrm{mol}$	^39^

Since the heat capacity of biological tissue strongly depends on tissue water content and is therefore temperature-dependent, it was also incorporated into the model, following the approach of Chen et al. (2013)^17^. The effective heat capacity, $C_\textrm{eff}(T)$, was defined as the sum of three components: $C_\textrm{t}$, $C_\textrm{w}$, and $C_\textrm{LH}$. Here, $C_\textrm{t}$ accounts for the tissue-specific heat capacity, $C_\textrm{w}$ represents the contribution from the tissue water, and $C_\textrm{LH}$ accounts for the energy required for the water phase change from liquid to vapor. Assuming 70% tissue water content, 30% of the literature-based effective heat capacity was attributed to $C_\textrm{t}$, while 70% was attributed to $C_\textrm{w}$. The latent heat component, $C_\textrm{LH}$, was defined as the product of the latent heat of water ($2.26~\textrm{MJ}/\textrm{kg}$) and the first derivative of *W*(*T*) from Equation (16).

Consequently, a temperature increase during tissue coagulation results in a reduction of tissue water content, as described by Equation (16), leading to a decrease in the effective heat capacity while storing significant energy for the phase transition. As a result, the effective heat capacity has its maximum at $100~^{\circ }\textrm{C}$, which is caused by the enormous amount of energy that must be applied for the phase transition of tissue water, and consequently decreases significantly as the change in relative water content drops^40^. All other material parameters for biological tissue were considered temperature-independent in the simulations. The material properties of the tissue, which were considered to be liver tissue in this analysis, and of the stainless steel electrode are summarized in Table 1.

### Damage model

In monopolar tissue coagulation, the goal is to induce targeted thermal damage to biological tissue at the application site in order to achieve hemostasis through Joule heating. For predicting thermal tissue necrosis, the first-order Arrhenius equation has been established as a reliable damage model that describes a one-step irreversible damage process^41–44^. This equation characterizes the temperature dependence of the reaction rate constant and is used to model the rate at which thermal damage accumulates in the tissue. By interpreting the damage process as a cumulative reaction over time, the rate of change in damage is assumed to be proportional to the reaction rate constant. Consequently, the model accounts for both the applied temperature and the duration of exposure. The exponential relationship between temperature and exposure time is governed by tissue-specific kinetic parameters that describe the rate of thermal protein denaturation^45^. This irreversible process leads to thermal necrosis and is often associated with observable macroscopic changes, such as discoloration^46^.

The degree of tissue damage is described by the dimensionless damage parameter $\Omega (\tau )$, which represents the natural logarithm of the ratio between the initial viable (native) tissue *C*(0) and the remaining viable tissue fraction $C(\tau )$ at time $\tau$. The damage parameter is determined by integrating the damage rate ($\frac{d\Omega }{dt}$) over time. Accordingly, the damage integral is defined as

17 $$\begin{aligned} \Omega (\tau )= \ln \left\{ \frac{C(0)}{C(\tau )}\right\} =\int _0^{\tau } A_0 \cdot \exp \left( \frac{-E_{\textrm{a}}}{R\cdot T(t)}\right) \textrm{d}t, \end{aligned}$$

where $A_0$ is the frequency factor (characterizing the tissue’s sensitivity to thermal damage), $E_{\textrm{a}}$ is the activation energy for irreversible tissue damage (quantifying the temperature dependence of the damage process), and *R* is the universal gas constant (relating thermal energy to temperature on a molecular level). The damage integral $\Omega (\tau )$ thus provides a cumulative, dimensionless measure of thermal damage over time. When $\Omega$ reaches a value of 1, our analysis assumes that the tissue has become necrotic. At this point, approximately 63.2% of the viable tissue is assumed to be irreversibly damaged, which, according to Henriques (1947)^41^, corresponds to a second-degree burn.

To analyze the extent of necrosis, the necrotic tissue volume $\theta _{\textrm{V}}$ is determined. In our idealized 2D axisymmetric model, the geometry is described by the coordinate system ($r, z, \phi$), where $\phi$ represents the angular coordinate around the axis of symmetry (*z*-axis) with $\phi \in \left[ 0,2\pi \right]$. The necrotic volume is calculated by integrating over the region where the thermal damage is $\Omega (r,z)\ge 1$:

18 $$\begin{aligned} \theta _{\textrm{V}}= \int _{\Omega (r,z)\ge 1}^{}\textrm{dV}. \end{aligned}$$

Here, $\textrm{dV}$ denotes the 3D volume element, which is generated by the rotation of the 2D model around the axis of symmetry and is defined as $\textrm{dV}=2\pi r~\textrm{d}r~\textrm{d}z$.

### Finite element model, boundary and initial conditions

To analyze the problem under investigation, a 2D axisymmetric FE model of monopolar soft-coagulation was developed. In order to reduce the complexity of the real 3D problem, it was assumed that the ball electrode is positioned centrally and perpendicularly on a sufficiently large section of biological liver tissue. Both the electrode and the tissue were modeled as rotationally symmetric with respect to the electrode axis, thereby explicitly excluding any lateral tilt of the electrode. Furthermore, asymmetric anatomical features, such as irregular organ surfaces, boundary contours, and vascular structures were neglected in the model. As previously described, the liver tissue was assumed to be homogeneous and isotropic. These idealized assumptions and simplifications result in a rotationally symmetric contact condition between the electrode and the tissue, thereby enabling a generalized model that focuses specifically on the influence of the electrode-tissue contact area on Joule-induced heat generation, independent of individual anatomical variability.

**Fig. 1:**
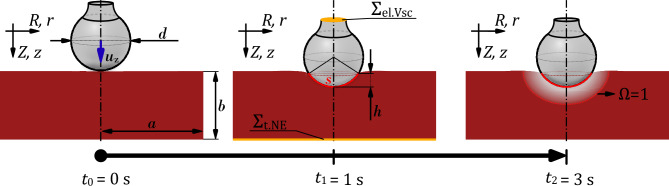
Schematic representation of the time-dependent simulation study of a monopolar soft-coagulation model. The figure shows the 3-second long coagulation process at three different points in time: First, at the initial state of the simulation (at $t_0$). Second, after a simulation time of 1 second (at $t_1$) and third, after a simulation time of 3 seconds (at $t_2$). Additionally, variables like the diameter of the electrode *d*, the height *b* and width *a* of the tissue block, the height of the sphere segment *h*, the circular arc *s* of the contact area, the applied voltage boundary conditions $\Sigma _{\mathrm {el.Vsc}}$ and $\Sigma _{\mathrm {t.NE}}$, as well as a schematic representation of the necrotic zone and its boundary at $\Omega = 1$ are shown.

To minimize the influence of artificial boundary conditions, the geometric dimensions of the tissue model were chosen to be notable larger than those of the electrode. The height and width of the tissue domain were defined in such a way that the electric and thermal field distributions within the tissue could be reasonably approximated as radially symmetric around the electrode axis. This assumption maintains the focal point of the analysis and its results on the primary interest: the impact of different contact sizes on Joule heating and the formation of thermal tissue necrosis. Thus, as depicted in Fig. 1, a conventional ball electrode with a diameter of 4 mm was modeled. The electrode includes a cylindrical shaft measuring 2 mm in diameter and 1 mm in height, with a rounded transition. The dimensions of the cylindrical tissue block were set to a height $b=1.5$ cm and a radius $a=1.5$ cm.

A time-dependent simulation of the monopolar HF soft-coagulation process was conducted over a total duration of 3 seconds. The simulation results were output with a temporal resolution of 0.1 s for the first second and 0.01 s for the remaining two seconds, enabling accurate resolution of the transient thermal and mechanical phenomena. The time-step size was automatically controlled by the solver based on a relative tolerance of 0.001, thereby ensuring numerically stable and accurate integration of the governing equations. During the initial phase of the simulation (first second), the mechanical contact between the ball electrode and the biological tissue block was established by prescribing a ramped displacement of the electrode in the axial z-direction, denoted by the displacement vector $\textbf{u}$. To investigate the influence of different electrode-tissue contact areas on Joule heating, this displacement was varied across seven values using a parametric sweep from $u_{\textrm{z}}=0.5~\textrm{mm}$ to $2~\textrm{mm}$ in increments of 0.25 mm. The fully coupled Augmented Lagrangian contact method^26^ was employed to model the contact, allowing robust handling of nonlinearities such as large deformation and frictionless, surface-to-surface interactions.

With respect to mechanical boundary conditions, the lower surface of the tissue block was constrained using a roller support, which permitted displacement in the radial r-direction while restricting motion in the axial z-direction. This condition reflects the assumption of a mechanically stable tissue foundation and ensures global static equilibrium during electrode indentation. All other external boundaries of the tissue domain, including lateral and upper surfaces, were left unconstrained, allowing the soft tissue to deform freely in response to electrode-induced loading. This setup accommodates the compliant and deformable nature of biological soft tissue and provides a sufficiently realistic approximation of local tissue deformation and surface curvature for the purposes of this study.

For the period $1\le t \le 3~\textrm{s}$, a RMS voltage of 98 V was applied to the upper surface of the electrode ($\Sigma _{\mathrm {el.Vsc}}$, see Fig. 1). The neutral electrode (ground) was defined at the lower boundary of the tissue block ($\Sigma _{\mathrm {t.NE}}$) with a potential of 0 V. The right boundary of the tissue block, along with parts of the upper boundary of the tissue and the non-contacting surfaces of the electrode, were treated as electrically insulating.

Since this analysis is based on an ex vivo application scenario, all exposed surfaces of both the electrode and the liver tissue that were subjected to convective cooling toward the ambient temperature $T_{\textrm{amb}}$ via a heat flux normal to the respective surface. In accordance with the axisymmetric modeling approach, the convective heat exchange with the environment was idealized and assumed to be spatially uniform across these surfaces. A convective heat transfer coefficient of $25~\textrm{W}/(\textrm{K}\cdot \textrm{m}^2)$ and an ambient temperature of $T_{\textrm{amb}} = 21~^{\circ }\textrm{C}$ were applied, in accordance with values reported in the literature^47^. Given the short duration of energy application and the highly localized nature of the resulting temperature increase within the biological tissue, the overall cooling effect induced by convection at the free surfaces was assumed minimal. Consequently, thermal radiation was neglected from the model.

At the initial time $t_0$, the electrode and tissue block were just barely in contact without any forces acting between the surfaces. Consequently, there were no internal stresses, displacement, or acceleration fields in either geometry at this time. Furthermore, the electrical potential in all geometries was initialized to 0 V. At $t_0$, the temperature of the electrode was set to $19~^{\circ }\textrm{C}$, while that of the tissue block was set to $21~^{\circ }\textrm{C}$.

The choice of an appropriate mesh was a critical factor for this application. The highly nonlinear behavior of the system, resulting from the implementation of temperature-dependent tissue parameters and the damage model predicting necrotic tissue volume, necessitated an accurate meshing strategy. As highlighted in Busch et al. (2024)^48^, an adequately fine mesh in the critical tissue regions was essential for accurately determining the necrotic volume. Specifically, the mesh in the contact region and its vicinity needed to be fine enough to capture the strong nonlinear phenomena accurately. Therefore, for enhanced mesh control, the upper boundary of the tissue block was divided into two regions, starting from the axis of symmetry with a radius of 2 mm. This allowed for the use of a finer mesh in the contact area, where Joule heating and thermal tissue damage occur, while using a coarser mesh in less critical regions.

The COMSOL Mesh Generator was employed to generate the mesh, with manual constraints on the maximum element size and growth rate. The maximum mesh sizes for the electrode and tissue area were set so 1.29 mm and 0.75 mm, respectively. The element size at the electrode-tissue interface, where contact occurs, was limited to a maximum of $25~\mu \textrm{m}$. The element growth rate of the tissue block was set to 1.03. Only triangular elements were used for meshing. The resulting model consisted of 18344 elements. The fully coupled linear direct solver PARDISO, provided by COMSOL Multiphysics®^20^, was utilized to solve the problem.

### Relation between displacement and contact area

As previously described, the simulation begins by solving a mechanical contact problem induced by the displacement of the ball electrode. This displacement is represented by the displacement vector $\textbf{u}$ in a 2D axisymmetric coordinate system $(r,z,\phi )$. Since the electrode is displaced exclusively along the axial z-direction, it is described in our analysis by the component $u_{\textrm{z}}$. Due to the axisymmetric nature of the model, the resulting contact area $M_{\textrm{cs}}$, which forms after a given electrode displacement $u_{\textrm{z}}$, cannot be directly extracted from the simulation results. Instead, it must be derived from a 2D simulation quantity. Therefore, the following section briefly outlines how the 3D contact area can be calculated from the 2D output variable $s(u_{\textrm{z}})$ using elementary trigonometric relationships. The variable $s(u_{\textrm{z}})$ represents the arc length of a circular segment and corresponds to the distance between the outermost contact points of the electrode with the tissue surface in the 2D axisymmetric model (see Fig. 1).

The actual contact area that is relevant for our thermal analyses is determined by the mechanical interaction between the ball electrode and the deformable tissue. As this interaction results in a nonlinear contact geometry, the contact area cannot be derived using simple geometric assumptions alone. Instead, it corresponds to the lateral surface area of a spherical segment in 3D space and is defined as $M_{\textrm{cs}}(u_{\textrm{z}}) = \pi d h(u_{\textrm{z}})$, where *d* denotes the diameter of the ball electrode and *h* represents the segment height. To evaluate $M_{\textrm{cs}}(u_{\textrm{z}})$, it is therefore necessary to determine the segment hight of a circular segment in 2D representation. Due to the nonlinear mechanical properties of soft tissue, the actual segment height $h(u_{\textrm{z}})$ is typically smaller than the prescribed axial displacement $u_{\textrm{z}}$. Accordingly, a geometric relationship must be established between the arc length $s(u_{\textrm{z}})$ and the segment height $h(u_{\textrm{z}})$ in the 2D setting. This relationship is derived using basic trigonometric principles by

19 $$\begin{aligned} h(u_{\textrm{z}})=\frac{d}{2} \left( 1-\textrm{cos}\left( \frac{s(u_{\textrm{z}})}{d}\right) \right) . \end{aligned}$$

**Fig. 2:**
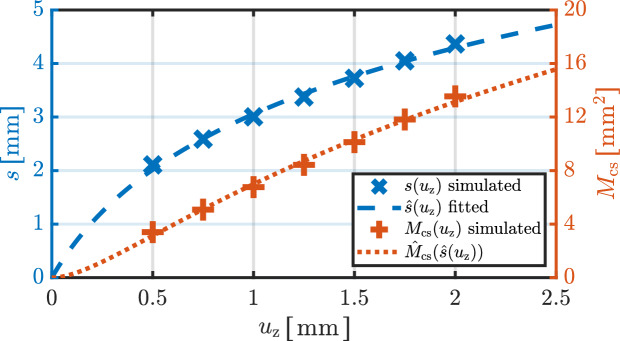
Simulated relationship between the electrode displacement $u_{\textrm{z}}$ and the resulting arc length $s(u_{\textrm{z}})$ as well as the corresponding contact area $M_{\textrm{cs}}(u_{\textrm{z}})$. The seven data points for $s(u_{\textrm{z}})$ were extracted from the mechanical contact simulation and used to calculate the associated contact areas $M_{\textrm{cs}}(u_{\textrm{z}})$. The fitted curves $\hat{s}(u_{\textrm{z}})$ and $\hat{M}_{\textrm{cs}}(u_{\textrm{z}})$ are shown for illustrative purposes only and were not used for further analytical approximations.

To determine $M_{\textrm{cs}}(u_{\textrm{z}})$, the arc length $s(u_{\textrm{z}})$ was extracted from the simulation results for the electrode displacements ranging from 0.5 mm to 2 mm in increments of 0.25 mm. Due to the combined influence of the spherical geometry and the nonlinear tissue deformation, $s(u_{\textrm{z}})$ exhibits a saturation behavior with increasing displacement. To illustrate this behavior, a logarithmic regression was applied to the eight known values of $s(u_{\textrm{z}})$, resulting in the function $\hat{s}(u_{\textrm{z}})= 2.2131 \cdot \textrm{ln} \left( 1 + 2.978 u_{\textrm{z}} \right)$ with a coefficient of determination of $R^2=99.8\%$. Based on this regression and the geometric relationships described above, the corresponding nonlinear approximation of the contact area $\hat{M}(u_{\textrm{z}})$ was derived. The resulting relationship is illustrated in Fig. 2.

It should be emphasized that the regression curves are provided solely for illustrative purposes to visualize the trend of the nonlinear relationship. It is not used as a basis for any further modeling assumptions or analytic approximations.

## Results

**Fig. 3:**
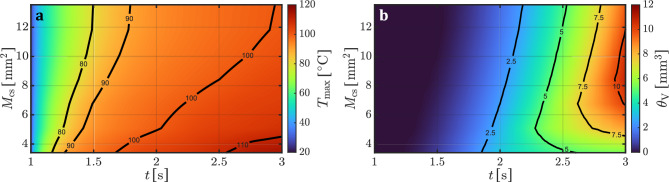
Simulated (**a**) maximum tissue temperature $T_{\textrm{max}}(t, M_{\textrm{cs}})$ and (**b**) necrotic tissue volume $\theta _{\textrm{V}}(t, M_{\textrm{cs}})$ as a function of application time *t* and electrode to tissue contact area $M_{\textrm{cs}}$. Due to the voltage activation at the simulation time $t=1~\textrm{s}$, both graphs show the development of $T_{\textrm{max}}$ and $\theta _{\textrm{V}}$ from this time until the end of the application time ($t=3~\textrm{s}$).

Figure 3a and b illustrate the progression of the maximum tissue temperature, $T_{\textrm{max}}$, and the necrotic tissue volume, $\theta _{\textrm{V}}$, as a function of the application time *t* and the electrode-tissue contact area $M_{\textrm{cs}}$. These surface plots utilize color gradients to visualize the development of maximum tissue temperature (Fig. 3a) and the formation of thermal necrosis (Fig. 3b) in response to increasing contact area and exposure time. The results clearly highlight the substantial influence of the contact area on both the thermal response of the tissue and the extent of necrosis formation during monopolar HF soft coagulation.

A detailed examination of the temperature trend in Fig. 3a reveals that as the contact area increases, the accumulation of heat in the tissue becomes significantly slower. Although the threshold of 80 $^{\circ }\textrm{C}$ is reached within the first 0.5 seconds across all simulated contact areas, the subsequent rate of temperature rise is inversely related to the contact area. This trend is further reflected in the position and spacing of contour lines for 90 $^{\circ }\textrm{C}$, 100 $^{\circ }\textrm{C}$, and 110 $^{\circ }\textrm{C}$.

For instance, with the smallest contact area of $3.4~\mathrm {mm^2}$, the tissue reaches 100 $^{\circ }\textrm{C}$ after approximately 0.68 seconds and exceeds 110 $^{\circ }\textrm{C}$ after 1.56 seconds. In contrast, with a moderate contact area of $8.42~\mathrm {mm^2}$, the threshold value of 100 $^{\circ }\textrm{C}$ is only reached after 1.5 seconds and then rises to a final temperature of 103.6 $^{\circ }\textrm{C}$. With the largest contact area of $13.54~\mathrm {mm^2}$, the tissue reaches 100 $^{\circ }\textrm{C}$ after 1.94 seconds, followed by a slight increase to 100.3 $^{\circ }\textrm{C}$ by the end of the application.

The influence of contact area on thermal behavior is also evident in Fig. 3b, which shows the progression of necrotic volume. Larger contact areas generally result in a greater extent of necrosis. Notably, there is a temporal delay before the onset of visible tissue damage, which correlates with the temperature contour of 80 $^{\circ }\textrm{C}$ seen in Fig. 3a. Thereafter, the necrosis volume increases gradually over time. Although the smallest contact area ($3.4~\mathrm {mm^2}$) leads to the earliest onset of tissue damage, the increase in necrotic volume is limited and plateaus relatively early. Specifically, a saturation-like behavior begins after approximately 1 second of voltage application. A similar trend is observed for contact areas of $5.08~\mathrm {mm^2}$ and $6.76~\mathrm {mm^2}$, with saturation occurring around 1.5 and 1.8 seconds after voltage application, respectively.

**Fig. 4:**
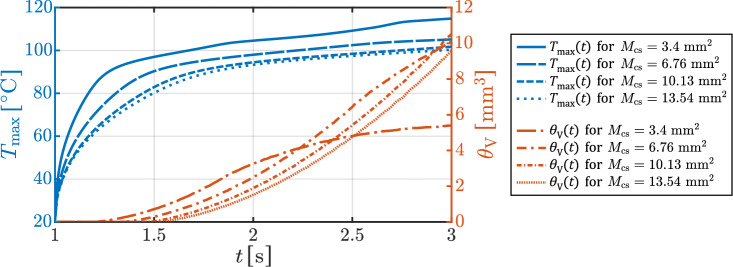
Simulated maximum tissue temperature $T_{\textrm{max}}(t)$ and tissue necrosis volume $\theta _{\textrm{V}}(t)$ as a function of the application time *t* at four different contact areas $M_{\textrm{cs}}$.

In contrast, from a contact area of $8.42~\mathrm {mm^2}$ onwards, the necrotic volume continues to increase steadily throughout the application time without evidence of saturation. This is reflected by the decreasing spacing between contour lines representing necrosis volumes of $2.5~\mathrm {mm^3}$, $5~\mathrm {mm^3}$, $7.5~\mathrm {mm^3}$, and $10~\mathrm {mm^3}$. The results indicate that slower heating, resulting from a larger contact area, promotes the formation of more extensive necrosis.

The development of necrotic volume appears to follow a logistic growth behavior, characterized by a delayed onset, a subsequent acceleration (inflection point), and an eventual transition toward saturation. This behavior is further illustrated in the line plots of Fig. 4, which display the maximum tissue temperature and the volume of thermal tissue necrosis over time for four representative contact areas: $3.4~\mathrm {mm^2}$, $6.76~\mathrm {mm^2}$, $10.13~\mathrm {mm^2}$, and $13.54~\mathrm {mm^2}$. The influence of the contact area is again apparent, as $M_{\textrm{cs}}$ increases, both the inflection point and growth constant of the logistic curve shift along the temporal axis, while the asymptotic necrosis volume increases significantly. For the two larger contact areas ($10.13~\mathrm {mm^2}$ and $13.54~\mathrm {mm^2}$), there is no indication of saturation by the end of the simulation.

**Fig. 5:**
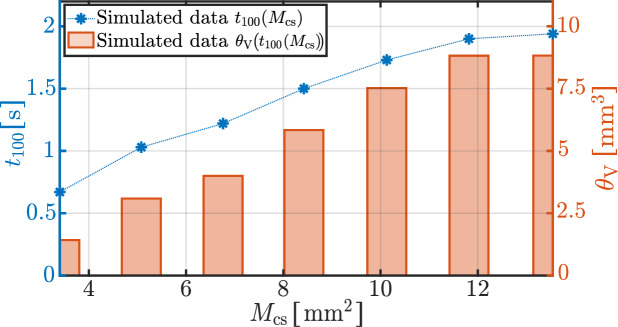
Shown is the simulated time at which the tissue temperature has reached 100 $^{\circ }\textrm{C}$ as a function of the contact areas $M_{\textrm{cs}}$. Furthermore, the extent of necrosis at the time points $t_{100}$ is indicated by the bars at the corresponding contact areas $M_{\textrm{cs}}$.

The moment at which the tissue reaches a temperature of $100~^{\circ }\textrm{C}$, denoted by $t_{100}$, is of particular interest, as it marks the onset of nonlinear processes related to water evaporation. Figure 5 quantifies the dependence of $t_{100}$ on the contact area. The blue dotted line (left y-axis) shows linearly interpolated values of $t_{100}(M_{\textrm{cs}})$, while the red bars (right y-axis) indicate the corresponding necrotic volumes at that time. An increase in contact area from $3.4~\mathrm {mm^2}$ to $6.76~\mathrm {mm^2}$ (corresponding to an increase in the contact area of $98.6\%$) delays $t_{100}$ by $82.1\%$ and results in a $180.3\%$ increase in necrosis volume. Further increasing the area to $10.13~\mathrm {mm^2}$ (a $49.8\%$ increase in contact area) delays $t_{100}$ by additional $41.8\%$, with an $88.2\%$ increase in necrosis volume. Finally, increasing $M_\textrm{cs}$ to $13.54~\mathrm {mm^2}$ (a $33.7\%$ increase in contact area) leads to a further $12.1\%$ delay in $t_{100}$ and a $17.3\%$ increase in necrosis. Additionally, $6.76~\mathrm {mm^2}$ can be compared to $13.54~\mathrm {mm^2}$ (a $100.3\%$ increase) because this pair, like $3.4~\mathrm {mm^2}$ and $6.76~\mathrm {mm^2}$, represents an approximately twofold increase in contact area. In this case, the heating process is slowed by $59\%$, and the necrosis volume increases by $120.8\%$. These results indicate that beyond a certain threshold (approximately $10.13~\mathrm {mm^2}$), further increases in contact area lead to diminishing returns in terms of heating efficiency.

**Fig. 6:**
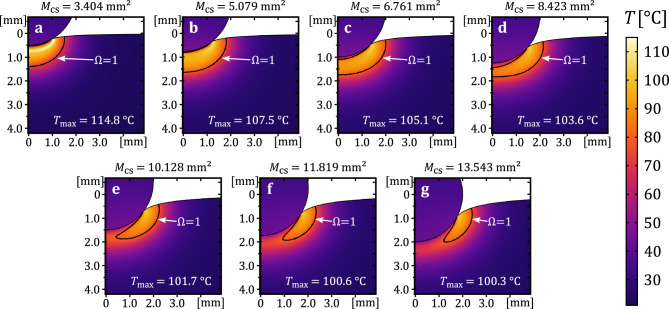
Simulated spatial temperature distribution at the contact location at the end of the application time ($t=3~\textrm{s}$) for the seven different contact areas. The temperature field is shown in the color scale from dark blue for $21~^{\circ }\textrm{C}$ to light yellow for $115~^{\circ }\textrm{C}$. The necrosis boundary is illustrated in the temperature field and given by a damage parameter of $\Omega =1$.

**Fig. 7:**
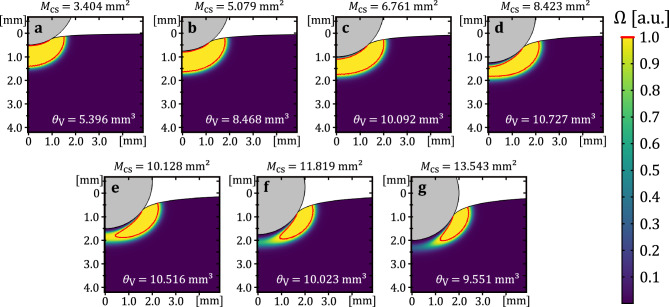
Simulated spatial tissue damage distribution at the contact location at the end of the application time ($t=3~\textrm{s}$) for the seven different contact areas. The state of tissue damage is shown in the color scale from dark purple as healthy tissue to yellow for necrotic tissue. The necrosis boundary is illustrated in the damage field by a red line and is given by $\Omega =1$.

Figures 6 and 7 illustrate the spatial distributions of temperature and the state of tissue damage in proximity to the contact surface at the seven distinct contact areas at the end of the 2-second voltage application period ($t=3~\textrm{s}$), respectively. All graphs also illustrate the boundary at which the damage parameter, denoted by $\Omega$, attains the value of 1. In Fig. 6, the heating process is seen to vary with contact area, ranging from complete to still progressing. For instance, Fig. 6a shows the development of a high-temperature region (up to 115 $^{\circ }\textrm{C}$) beneath the electrode. Given the temperature-dependent tissue properties implemented to account for evaporation effects at 100 $^{\circ }\textrm{C}$, it is evident that this region is highly resistive. In the corresponding damage plot in Fig. 7a, a complete necrotic region beneath the electrode is observed, consistent with the saturation exhibited in Fig. 4.

In Fig. 6b, the high-temperature region begins to form radially from the edge of the contact area towards the axis of symmetry. The associated necrosis plot in Fig. 7b shows a closed necrotic region, indicating that damage is essentially complete. The closed necrotic region is subsequently also referred to as a necrotic plug. At $M_{\textrm{cs}}=6.76~\mathrm {mm^2}$, Figs. 6c and 7c still show that a closed, continuous necrotic plug has formed. However, a small, undamaged region remains near the axis of symmetry at the contact interface. This incomplete necrosis becomes more pronounced with increasing contact area, as shown in subfigures (d) through (g).

Figure 6d-g further show that the maximum temperature no longer increases significantly and is just over 100 $^{\circ }\textrm{C}$. Instead, it can be concluded from the figures that the heat front continues to propagate laterally from the outer edge of the contact region towards the center. This leads to a gradual expansion of the necrosis in the corresponding damage distributions (Fig. 7d-g) and the formation of a necrotic plug, while the maximum tissue temperature remains almost constant. In combination with Fig. 4, these findings confirm that the necrotic region beneath the electrode has not yet fully developed at the time when $T_{\textrm{max}}=100~^{\circ }\textrm{C}$, and therefore, a complete necrotic plug is not yet present.

## Discussion

In this study, a multiphysics simulation model integrating mechanical, thermal, and electrical aspects is employed to analyze the effects of electrode-tissue contact area on thermal dynamics and necrosis formation during monopolar HF soft coagulation of soft tissue. The knowledge gained from this research should lead to a better understanding of the processes involved and to further development of control applications. The results demonstrate the importance of the contact surface for the electrocoagulation process through its influence on the heating dynamics and the formation of necrotic tissue volume.

A small contact area between the electrode and the tissue was determined to result in higher current densities and high contact resistance at the contact site. This phenomenon leads to a localized but high energy deposition and therefore, to a rapid temperature development within a smaller tissue volume. Graphical results, such as Figs. 3, and 4, illustrate that a smaller contact area not only accelerates temperature rise but also limits necrotic volume early in time. Conversely, an increase in contact area has been demonstrated to reduce contact resistance, current density, and thus energy deposition in a larger tissue volume. This slows the heating process (see Fig. 5), delays the critical boiling point, and delays the formation of high-resistance tissue. Consequently, there is a significant increase in necrotic tissue volume due to enhanced heat spread.

In addition to the influence of the contact area on parameters such as contact resistance and current density, an increase in contact area results in energy deposition in a greater volume of tissue, thus forming a larger necrotic tissue volume. To counteract the formation of a significantly larger necrosis volume at a larger contact area, the applied voltage could be increased, which would ultimately result in a stronger electrical field and higher current densities at the contact side. This results in accelerated temperature development in the tissue near the contact surface. The more pronounced and rapid heating at the contact surface leads to superficial necrosis, while the necrosis formation in deeper tissue areas is limited due to the high resistive nature of superficial necrotic tissue at the contact side, which hinders current flow and thus Joule heating in deeper tissue areas.

A detailed analysis of the temperature dynamics at the various contact areas indicates the presence of an inhomogeneous electrical field at the contact side. This leads to a maximum temperature development at the edges of the electrode-tissue contact area (see Fig. 6). This preferential heating initiates consequently necrosis in a ring-shaped pattern, as also observed in one of our earlier studies^49^. Over time, the necrotic and resistive tissue at the contact edges concentrates current flow in the non-necrotic region beneath the electrode. With continued energy application, necrosis extends radially from the edges towards the electrode center, eventually forming a necrotic plug that severely restricts current flow in this region. This progression is characterized initially by slow necrotic volume growth, followed by a rapid, quadratic increase as energy deposition concentrates more intensely on the healthy tissue beneath the contact area (see Fig. 4). Therefore, a delay is expected between the onset of rapid necrotic volume growth and the peak temperature observed. Once a continuous necrotic layer forms, necrotic volume growth decelerates, resulting in an almost stable volume as observed in our results.

Additionally, it can be assumed that for larger contact areas, the concentrated electric field at the contact edges results in a more pronounced temperature gradient across the contact site. The temperature dependence of electrical conductivity, in conjunction with the consideration of tissue water evaporation at the boiling point of water, introduces further nonlinear effects that influence the heating process and necrosis formation. This temperature gradient along the contact area causes necrosis to form predominantly at the contact edges. In contrast, it has been demonstrated that the application of a constant voltage in relation to smaller contact areas leads to a localization of the current density, thereby reducing the inhomogeneity at the contact surface.

Further insights on the development of tissue necrosis, starting with a ring-shaped structure and ending with a necrotic plug, can be deduced from Fig. 7. The simulated spatial distribution of tissue necrosis shows that a continuous necrotic plug has not yet formed on the contact surface when a temperature of $100~^{\circ }\textrm{C}$ is reached. This is a significant finding that should be emphasized. This underlines the inhomogeneous heating process at the contact surface during coagulation using a ball electrode. Thus, regardless of the contact surface, a temperature of $100~^{\circ }\textrm{C}$ is present at the edges of the contact surface in the tissue. At this point in time, the reached temperature field only leads to localized tissue necrosis. This necrosis appears to extend to nearly half of the tissue volume at the contact surface. Consequently, a complete coagulation has not yet been achieved, and there is an incomplete necrotic plug below the contact surface.

Prolonged exposure of the tissue to energy now leads not only to a further spread of necrosis across the contact surface but also to a continued increase in temperature at the edges of the contact area. We hypothesize that this process promotes the occurrence of a so-called popcorn effect in the tissue, which refers to a micro-explosion caused by a rapid increase in internal pressure. In other areas of research, such as the ablation of heart patients, this effect is reported and described as a steam pop^50,51^. This phenomenon arises when gaseous tissue water accumulates due to an excessively rapid and intense heating process, preventing the gas from escaping^52^. Once the pressure exceeds a critical threshold, the tissue undergoes a sudden rupture. Consequently, the necrotic tissue zone is disrupted, potentially leading to renewed bleeding or further undesirable damage to healthy tissue. The already necrotic tissue edges of the contact area make it more difficult for water vapor to escape from the tissue and become trapped in the tissue below the electrode. As electrosurgical-induced coagulation progresses, the pressure in the tissue below the contact side increases due to further evaporation of tissue water, promoting the popcorn effect.

While our study aims to simulate the effect of monopolar HF soft coagulation and gain insights for a deeper comprehension of the application, which can then be used for future development of control applications, it should be noted that the simplifications and assumptions made may lead to deviations from real applications. However, all considerations in this study are fundamentally based on physical principles as well as established and, in part, validated assumptions. For this reason, it is assumed that while deviations from real-world conditions are possible, the general findings of our simulation regarding the influence of the electrode-tissue contact area on the temperature dynamics and the necrosis formation provide a sufficiently accurate representation of reality for our intended purposes. Nevertheless, in the following, we will briefly discuss the potential influence factors and limitations of our model.

The reduction of the inherently 3D problem to a 2D axisymmetric FE model necessitates several assumptions and simplifications that may introduce deviations from the behavior observed in realistic 3D ex vivo scenarios. In particular, the model assumes that the ball electrode is placed perfectly centrally and perpendicularly on the tissue domain, resulting in a homogeneous, radially symmetric current distribution about the axis of rotation. As a consequence, any tilting or lateral displacement of the electrode (conditions that may occur in real-world applications) cannot be represented within this modeling framework. However, given the spherical geometry of the electrode, it is reasonable to assume that minor inclinations (i.e., significantly less than $90^{\circ }$) do not affect the contact characteristics or the electrical behavior, provided that the displacement occurs orthogonally to the tissue surface. In such cases, the contact area remains effectively unchanged, and the axisymmetric approximation remains valid.

The simplification of the application into a 2D axisymmetric model is particularly justified in this study, especially considering the use of a ball electrode. This type of electrode represents the most commonly used design in monopolar HF soft coagulation, as it is particularly well-suited for localized, deep, and point-specific hemostasis. Although the diameter of ball electrodes may vary depending on the specific surgical context, the intended clinical effect of HF soft coagulation remains fundamentally the same. Therefore, the results presented in this study can be considered generally applicable across a range of commonly used ball electrode sizes.

Beyond this, the present analysis highlights the critical importance of the electrode-tissue contact area in HF surgical applications. This parameter has a decisive influence on the extent of energy deposition into the tissue and thus must be carefully considered in future simulation studies. The findings underscore that even within a simplified geometric framework, variation in contact area can lead to significant changes in the heating dynamics and the extent of tissue damage. It should be noted, however, that other, more complex electrode geometries, such as cylindrical roller electrodes (commonly used in laparoscopic procedures for superficial tissue coagulation), differ substantially in their intended clinical outcome and mode of application. Consequently, these electrode types require separate modeling approaches. Nonetheless, the methodological approach of this work, such as the multiphysics coupling and the modeling of current-induced heat generation along with the resulting thermal tissue response, are not inherently limited to axisymmetric geometries. These elements can be transferred and extended to 3D models involving more complex electrode shapes.

Additionally, the 2D axisymmetric approach inherently assumes an idealized, flat tissue surface, which does not fully reflect anatomical realities, where tissue surfaces are often irregular and structurally complex. While these features could be incorporated in a full 3D simulation, doing so would require case-specific geometrical modeling and is thus outside the scope of this generalized analysis.

The most significant limitation introduced by the 2D axisymmetric formulation is the implicit assumption of perfect axial alignment between the active electrode and the neutral electrode, as well as to the boundaries of the tissue domain. A 3D model could explicitly account for asymmetries in the electrode arrangement or anatomical context, which may lead to non-uniform current paths, asymmetric heat distribution, and consequently uneven necrosis patterns. Nevertheless, due to the relatively large size of the tissue domain compared to the electrode dimensions, it is assumed that minor asymmetries and inhomogeneities in a realistic scenario would only have a marginal impact on the key outcome of interest.

The tissue properties are generally assumed to be homogeneous and isotropic^22–25,53^, despite the possibility of anisotropy^54^. Factors such as fiber structure and orientation, as well as the presence of vascular structures or local variations in conductivity and perfusion, have been demonstrated to influence the propagation of the coagulation zone, thereby causing it to become asymmetric^55^. However, these factors can be neglected for an initial general investigation of the influence of the electrode-tissue contact area on the heating dynamics and necrosis formation. Furthermore, the behavior of temperature-dependent tissue parameters is predominantly determined by measurements and assumptions, as these effects are challenging to quantify or verify through direct measurements. The utilized values in this study were primarily derived from other research groups, as outlined in the Methods section. The extent to which our assumptions align with reality can only be determined through the conduction of practical ex vivo validation tests, which are planned for the future.

The hyperelasticity of the tissue is modeled using one of the simplest hyperelastic material models, which relies solely on Young’s modulus and Poisson’s ratio. However, it should be noted that this approach may result in discrepancies when dealing with large strains (>30%)^56^. In the domain of tissue engineering, alternative hyperelastic material models, such as the Mooney-Rivlin or Odgen model, are frequently employed and could more realistically represent the deformation behavior of biological tissue^22^. Nevertheless, these models require additional material-specific parameters, consequently exhibiting higher complexity and reduced numerical stability^56^. For instance, depending on the selected Odgen model type (e.g., two-term, three-term, or four-term model), four, six, or eight parameters are necessary to define the material behavior^57^. While the implementation of a more sophisticated model could offer a more precise depiction of the deformation in the mechanical contact problem, it would not lead to any alteration in the fundamental findings discussed previously. Specifically, while the relationship between the displacement of the electrode and the resulting electrode-tissue contact area would change, Joule heating and thermal necrosis formation at specific contact areas would remain unaffected.

The use of the Mikic elastic correlation for modeling the electrical contact conductance $h_{\textrm{c}}$ introduces several simplifications. Originally developed for isotropic, rough, linear-elastic solids, the Mikic model assumes elastic deformation of surface asperities and statistically homogeneous roughness^30^. Consequently, it does not capture the full complexity of hyperelastic, heterogeneous biological soft tissue or changes in contact behavior due to temperature effects, dehydration, or surface fluid films. Friction and local anisotropies are likewise neglected. Therefore, the Mikic correlation was applied in this study as a macroscopic approximation of the electrical contact behavior. Although the tissue is modeled as hyperelastic, the Mikic model relies on an effective local linearization of the contact behavior to approximate the contact modulus. The resulting conductance $h_{\textrm{c}}$ was then determined from the nonlinear local contact pressure and the estimated material parameters. This approach allows the contact behavior to be incorporated in a computationally efficient manner, focusing on the influence of the electrode-tissue contact area on Joule-induced heating. Although relatively low contact pressures occur in our case (about $3~\textrm{kPa}$), there is a potential possibility that a significant local deformation of the tissue at the electrode contact point may lead to an underestimation of the contact conductance at large electrode displacements. Consequently, this could lead to deviations in the heat generation, which necessitates further investigation. While more detailed multiphase or poroelastic contact models might yield greater accuracy, their application is currently limited by the lack of validated constitutive parameters and contact properties for biological tissue under high-frequency electrical loading. As such, the Mikic-based approximation offers a tractable and physically plausible engineering solution for the specific goal of this research.

A critical factor for the successful application of HF current induced soft coagulation in achieving effective hemostasis is the targeted, superficial dehydration of tissue. This process involves the evaporation of tissue water during heating, which results in localized tissue shrinkage at the electrode-tissue interface^58^. Small veins, arteries, and capillaries contract and seal as a part of the electrocoagulation process. However, this phenomenon of tissue shrinkage is not currently incorporated into the presented model but could be considered through the inclusion of the thermal expansion coefficient, $\alpha \ne 0$. The omission of tissue shrinkage in the model (i.e., $\alpha =0$) may lead to minor deviations in the predicted necrotic volume compared to real-world outcomes but does not change the influence of the contact area on necrosis formation.

Another limitation is that, in real-world surgical applications, the applied voltage is typically not held constant. Instead, the user selects the power level and the desired effect on the HF generator. Depending on the resistive properties of the tissue during the electrocoagulation process and the selected settings on the HF generator, the voltage is adjusted. This voltage control mechanism has not yet been implemented in the model, which may lead to differences in the predicted temperature rise compared to real-world applications. Although our investigation is based on well-established physical equations, the complex processes occurring within the tissue, combined with the control algorithms of HF generators, present significant challenges for accurate simulation. Real-world measurements could provide greater certainty to the simulation results. Despite the omission of power regulation in the simulation, the findings of this study offer significant insights into the effect that various contact surfaces of the electrode with the tissue have. Consequently, future research endeavors will facilitate the optimization of the generator control and the integration of a control application considering the contact area, thereby enabling the attainment of a desired coagulation zone.

The present analysis clearly demonstrates the significant influence of the electrode-tissue contact area on both the development of the maximum tissue temperature and the formation of the necrotic tissue volume. An increase in the contact area leads to an expansion of the coagulation volume. This phenomenon can be attributed to two primary factors. First, an increase in contact area results in a reduction in contact resistance and a reduced current density, which, in turn, slows down the electrocoagulation process while extending it over a larger tissue volume. Second, an increased contact area inevitably leads to energy deposition over a larger volume, thereby enlarging the coagulation zone. Consequently, it can be deduced that the contact area is a significant impact factor in electrocoagulation applications and should also be taken into account in any practical experiments. In particular, for experiments conducted under laboratory conditions, it is imperative to exercise caution during the experimental setup. This precaution will ensure that the influence of the power control function or any other control application can be demonstrated to be safe and effective.

From the perspective of a control application and in relation to our research objective, it can be concluded that the contact area plays a crucial role in HF current induced soft tissue coagulation, as it significantly affects the electrocoagulation process. However, the optimization of existing control algorithms or the development of a novel control feature for an HF generator appears to be a reasonable endeavor only below the coagulation saturation threshold. Once a necrotic plug has formed at the contact site, the subsequent propagation of necrosis is substantially hindered due to the high resistivity of the necrotic tissue. For reasons of safety, it is therefore recommended to refrain from a significant increase in the applied voltage at this point, in order to prevent the extent of undesirable tissue damage.

## Conclusion

This study presents a novel investigation into the impact of electrode-tissue contact area on thermal dynamics and necrotic volume formation during monopolar HF soft coagulation using a ball electrode. By employing a fully coupled mechanical-thermal-electrical FE model, we quantitatively demonstrated, based solely on first principles, the strong influence of mechanical contact conditions on the resulting heat distribution and tissue damage. The main contribution of this work lies in establishing a direct and mechanistically grounded relationship between electrode-tissue contact area and electrocoagulation outcomes, a factor that has been largely underrepresented in prior simulation studies of HF surgery. Our approach captures the dynamically evolving contact interface and its consequences for energy delivery and necrosis formation. These findings not only underscore the necessity of accounting for contact area in future simulation studies but also provide a robust theoretical basis for the development of advanced control mechanisms. Such mechanisms could enable real-time adaptation of surgical parameters based on electrode-tissue interaction, ultimately improving the safety, predictability, and efficacy of HF soft coagulation procedures.

## Data Availability

The datasets generated during and/or analyzed during the current study are available from the corresponding author on reasonable request.
